# 

**Published:** 1852-03

**Authors:** 


No. X. MARCH. Vol. VII.

### BUFFALO

### MEDICAL JOURNAL

AND

### MONTHLY REVIEW

OF

### MEDICAL AND SURGICAL SCIENCE.

EDITED BY AUSTIN FLINT, M. D.

BUFFALO:
PUBLISHED BY JEWETT, THOMAS & CO.
Commercial Advertiser Buildings.
1852

*Price $2.50 per annum . . . in Advance.*

